# Challenges and strategies in the multicultural sexual and reproductive healthcare encounter - a qualitative study on the perspectives of health professionals

**DOI:** 10.1186/s12978-026-02471-1

**Published:** 2026-10-14

**Authors:** Mia Appelbäck, Aubry Seery Wredle, Ayanthi Wickramasinghe, Birgitta Essén, Aje Carlbom

**Affiliations:** 1 Department of Women’s and Children’s Health, Uppsala University, Uppsala, Sweden; 2 Department of Obstetrics and Gynecology, Skåne University Hospital, Malmö, Lund, Sweden; 3 Department of Social Work, Faculty of Health and Society, Malmö University, Malmö, Sweden

**Keywords:** Sexual and reproductive health, Migrant health, Multicultural healthcare, Health professionals, Person-centered care, Intercultural communication, Clinical encounters, Pατieντ αuτoνoµy

## Abstract

**Background:**

Sexual and reproductive health (SRH) in Sweden is shaped by strong equity and gender-equality ideals, yet migrant patients continue to experience disparities in SRH outcomes. Social, cultural, and structural factors can complicate clinical encounters, potentially posing obstacles to equitable access. This study explored how SRH professionals experience and manage challenges in multicultural clinical encounters.

**Methods:**

Thirty‑one SRH professionals (midwives, nurses, physicians, social workers, sexologists, and nursing assistants) from youth and women’s health clinics in Skåne were interviewed using semi‑structured interviews. The material was analysed with inductive thematic analysis to identify recurring patterns of challenges and strategies in encounters with patients of foreign background.

**Results:**

Four interconnected themes were identified: (1) Navigating Structural Barriers in SRH Care; (2) Family Involvement, Power Dynamics and Autonomy; (3) Person-Centred Care Beyond Cultural Competence; and (4) Structural Opportunities and Constraints in Care. Providers described navigating individual preferences, family relationships, social and religious norms, and structural constraints. They reported using flexible, individualized strategies to support confidentiality, informed decision-making, patient autonomy, and access to care.

**Conclusions:**

SRH professionals described multicultural clinical encounters as shaped by intersecting individual, familial, social, and structural factors. Their accounts suggest that equitable SRH care should not rely on individual clinicians adapting to structural barriers. Institutional support for confidential communication, appropriate interpretation, patient-centred decision-making, protection from coercion, accessible health information, and privacy-sensitive digital systems is needed.

## Background

Sexual and reproductive health (SRH) is essential to individual well-being and broader social and economic development [1]. Its importance has been affirmed through international frameworks such as the 1994 International Conference on Population and Development [2] and the 2030 Agenda for Sustainable Development [3], which emphasize equitable access to SRH services for all. In Sweden, where healthcare is grounded in principles of equity and universal access, SRH services are expected to be inclusive and responsive to diverse needs [4–8]. In this study, the term *migrants* refers to foreign-born residents who migrated to Sweden. However, disparities persist, research shows that migrant women from low-income countries, particularly Sub-Saharan Africa, face higher risks of adverse pregnancy outcomes and maternal mortality [5, 8–12]. Beyond socioeconomic factors, communication barriers have also been identified [5, 10, 11]. While structural explanations are critical, further insight can be gained by exploring the perspectives of SRH professionals, whose experiences have been less investigated in previous research [11–13].

### Migration and SRH

Sweden is internationally recognized for its strong gender-equal policy system and the Swedish population expresses some of the world’s most liberal and egalitarian values [14]. At the same time, demographic changes driven by increased migration into Europe [3] have increased the proportion of foreign-born residents to around 20% of the population [15]. This group is highly diverse in terms of age, gender, and country of origin. However, many come from the MENA (Middle East and North African) countries in North- and East Africa where collectivist values are more predominant on a population level [14, 16, 17]. Research shows that individual characteristics influence values more than country of origin, and that religiosity is the strongest predictor of conservative attitudes [16–19]. This can influence attitudes toward gender roles and SRH, introducing new complexities in the clinical encounter, particularly as SRH providers tend to hold more liberal values than the general population. In this context, liberal social values refer to greater acceptance of gender equality, individual autonomy and self-determination, and diverse expressions of sexuality and sexual and reproductive choices [12, 20]. Providers’ approaches are shaped not only by personal values but also by institutional, ethical, and legal frameworks [11, 12]. Previous research on SRH and migrant health identified barriers such as language, religion, discrimination and limited health literacy [13, 21–23]. Yet fewer studies have examined how healthcare professionals themselves perceive and navigate these challenges [10, 21, 22].

### Healthcare providers perspectives

Previous research indicates that in efforts to support reproductive autonomy and gender equality, healthcare professionals may unintentionally prioritize interpretations of these principles that resonate with Swedish cultural norms when interacting with patients from more culturally or religiously conservative backgrounds [12, 23, 24]. This can create tensions, particularly when patients’ decisions are shaped by family or community pressures, placing some young women with migrant backgrounds at heightened risk of coercion related to contraception, perceived purity, or abortion [11]. This can leave providers in ethically complex situations as they navigate cultural sensitivity, structural constraints, and patient autonomy [11]. Attempts to act with cultural sensitivity with the aim of achieving culturally competent care, but without the necessary knowledge about the topic, may also lead to providers unintentionally stereotyping rather than practicing person-centred care [13, 23]. In this article, the term culturally competent care refers to an approach in which providers acknowledge and respond to the cultural, social, and linguistic contexts that shape patients’ health experiences. However, the concept of culture itself is complex. Culture is understood here as a dynamic and multifaceted construct, inseparable from beliefs, values, politics, economics, and broader structural conditions [25, 26]. Given this understanding, cultural competence has been criticized for being too essentialist, implying that clinicians can “learn” another person’s culture, despite its fluid, contextual, and heterogeneous nature.

Recent studies emphasize the need to respect migrant patients’ own views on gender equality, even when they diverge from Swedish norms [12–14, 23, 27]. Imposing dominant ideals may weaken the patient-provider relationship and reduce care accessibility [12, 13, 24]. Prior studies suggest that providers who adopt a reflective, person-centred, and respectful approach tend to build greater trust and deliver more effective care [11, 12, 28]. However, there is limited understanding of how these approaches are implemented in practice within multicultural healthcare settings [9, 10].

### Diversity among migrant populations

In undertaking a study which delves into the potential challenges that come with providing healthcare to migrants, it is crucial to acknowledge that there is considerable heterogeneity between different migrant groups in Skåne. The terms “migrants” and “foreign-born patients” encompass people with widely varying backgrounds, legal statuses, migration trajectories, health beliefs, and language proficiencies. Overly simplified generalizations must be avoided, as cultural and religious groups are internally diverse and individuals within the same perceived group may hold a wide range of views and values [16].

### Aim

The study aims to explore perceived challenges in clinical SRH encounters, with a focus on barriers and knowledge gaps that affect care accessibility and quality. Hence, the research questions are: What perceived challenges do SRH professionals face when caring for migrant patients with and what strategies and solutions to address these challenges do they report?

## Method

### Study design

This qualitative study is based on semi-structured interviews with SRH professionals in multicultural clinical settings in the region of Skåne, in southern Sweden. A qualitative approach was chosen to capture the nuanced experiences and reflections of healthcare providers in this field.

### Study setting

Participants were recruited from youth clinics and women’s health clinics in Skåne, primarily in and around Malmö, a city known for its long-standing cultural diversity [29, 30]. As of 2024, 38.5% of Malmö’s 362,000 residents were foreign-born, rising to 58% when including those with foreign-born parents, making it Sweden’s only majority foreign-background city [29]. This population includes commuters from nearby countries, refugees, and labour migrants, contributing to a rich mix of languages and cultural perspectives. However, areas with high proportions of foreign-born residents often are disproportionately affected by many social determinants of health such as lower socioeconomic status and barriers accessing healthcare [29]. Thus, Malmö provides a unique setting to explore how healthcare professionals engage with cultural diversity in clinical practice.

### Study participants

Thirty-one professionals were recruited (27 women, 4 men; aged 25–65), including 10 medical doctors (obstetrics/gynaecology), 15 nurses and midwives, 3 sexologists or social workers, and 3 nursing assistants. One-third were foreign-born. Recruitment initially used convenience sampling, whereby professionals known to one of the interviewers or working in the nearby area were invited to participate. Snowball sampling was also used, with additional participants recruited through recommendations from previously recruited participants. Finally, targeted sampling was used to broaden the range of experiences represented in the sample, particularly by recruiting more male professionals working at other women’s health clinics and youth clinics across the Region of Skåne.

### Data collection

Data were collected between September 2020 and March 2021. Due to restrictions related to the COVID-19 pandemic, interviews were conducted either at the participants’ workplaces or remotely using Zoom or Microsoft Teams. The interviews were carried out in Swedish or English with one interview conducted in Arabic, by the first author a medical doctor and another researcher a PhD candidate with a background in public health. Interviews lasted approximately 50 min on average, were audio-recorded, and subsequently transcribed verbatim. A semi-structured interview guide with open-ended questions was used to explore professionals’ experiences of providing SRH services to migrant patients [31]. The interviews covered experiences of working in culturally diverse healthcare settings, challenges and dilemmas encountered in SRH care, approaches used to manage these situations, and suggestions for improving healthcare professionals’ working conditions and practices. Following three initial pilot interviews, which were retained in the final dataset, minor modifications were made to improve the clarity of some questions and introduce additional follow-up questions. The interview guide was used flexibly to facilitate an open conversation while ensuring that the main areas of interest were addressed.

Recruitment and interviewing continued until the research team considered that sufficient thematic depth had been achieved. Specifically, saturation was considered reached when accounts became recurrent across participants and the final interviews did not contribute substantially new information regarding the challenges and dilemmas encountered or the strategies used to address them.

The same interviews and participants have been included in two previous publications [31, 32], but each study addressed a distinct research question and involved a separate analysis of the dataset. The first examined challenges and dilemmas in providing SRH services to migrants, strategies for navigating them, and the assumptions underlying providers’ accounts, using conceptual perspectives on person-centred care, power and knowledge, cultural competency, and cultural humility [31]. The second focused specifically on how the COVID-19 pandemic and associated changes in routines and social norms shaped providers’ experiences of intercultural clinical encounters [32]. In contrast, the present study involved a new inductive analysis focused on providers’ practical experiences of challenges and the strategies and solutions used in multicultural SRH encounters. Although some topics recur across publications because they arise from the same interviews, the four themes presented here were developed specifically for the present research question and analytical process. Where illustrative quotations have previously been reported, this overlap is acknowledged.

### Data analysis

The data were analyzed using an inductive thematic analysis informed by Braun and Clarke’s approach [33, 34]. Interviews were transcribed verbatim by members of the research team who conducted the interviews, as well as by a professional transcriber. The analysis for the present study was conducted manually without computer-assisted qualitative data analysis software. The analytical process was iterative. The members of the research team read and re-read the transcripts to become familiar with the data, taking notes and recording preliminary interpretations relevant to the study aim. The transcripts were then coded inductively, with codes generated from segments describing challenges, strategies, and perceived cultural differences in SRH care. As the analysis progressed, codes were compared across interviews, revised, and grouped according to recurring patterns and similarities. Related codes were subsequently organized into preliminary categories, which were further examined and refined into candidate themes and subthemes. The candidate themes were repeatedly reviewed against the coded extracts and the broader dataset to assess their coherence, relevance, and distinctiveness. Through this iterative process, the categories were progressively refined into the four final themes presented in the findings.

To strengthen the credibility and depth of the analysis, emerging categories and themes were reviewed and discussed within the multidisciplinary research team with backgrounds in gynecology, social anthropology, and sexual and reproductive health. These discussions were not used to establish coding consensus but rather to critically examine interpretations, challenge assumptions, and consider alternative explanations. The discussions informed the continued refinement of the themes and the final interpretation and presentation of the findings.

Illustrative quotations were selected after the themes had been developed and translated from Swedish into English when necessary. A generative AI tool [35] was used to support translation and language refinement, including suggestions for synonyms and stylistic improvements to pre-written text but was not used for data analysis or interpretation. All translated quotations were systematically checked against the original Swedish transcripts to ensure accuracy and preservation of meaning. Before being entered into the AI tool, quotations were de-identified by removing names and contextual details that could potentially identify participants, patients, or clinical settings.

### Researcher reflexivity

The research team brought diverse professional, disciplinary, and personal perspectives to the study. M.A. is a Swedish physician and PhD student with clinical experience working with migrant populations, while A.S.W. is a Swedish physician who had recently graduated at the time of the study. A.W. is a physician of Asian background, a migrant to Sweden, holds a PhD, and works in global health. B.E. is a Swedish gynecologist and professor of international maternal health with extensive clinical experience working with migrants and longstanding research experience in global health. A.C. is a Swedish associate professor of social anthropology with extensive research experience involving multiculturalism, religion, and migrants.

The researchers acknowledge that their professional, disciplinary, and personal positions may have influenced data collection and interpretation, particularly given the value-laden topics addressed in the study. Throughout the analytical process, interpretations and underlying assumptions were critically discussed within the multidisciplinary research team. The combination of clinical, global health, anthropological, and lived migration perspectives helped challenge assumptions and consider alternative interpretations.

## Results

The findings suggest that providers navigate the intersection of person-centred care and cultural competence when working with migrant patients. Across the material, clinicians describe how they balance patients’ individual needs, risks, and autonomy with an understanding of the cultural and social structures shaping their lives. This interplay becomes visible through four interconnected themes that together illustrate how providers manage barriers, negotiate family dynamics, and adapt care in ethically complex situations. The model below summarizes these themes and highlights the core dimensions that structure the results.

**Fig. 1:**
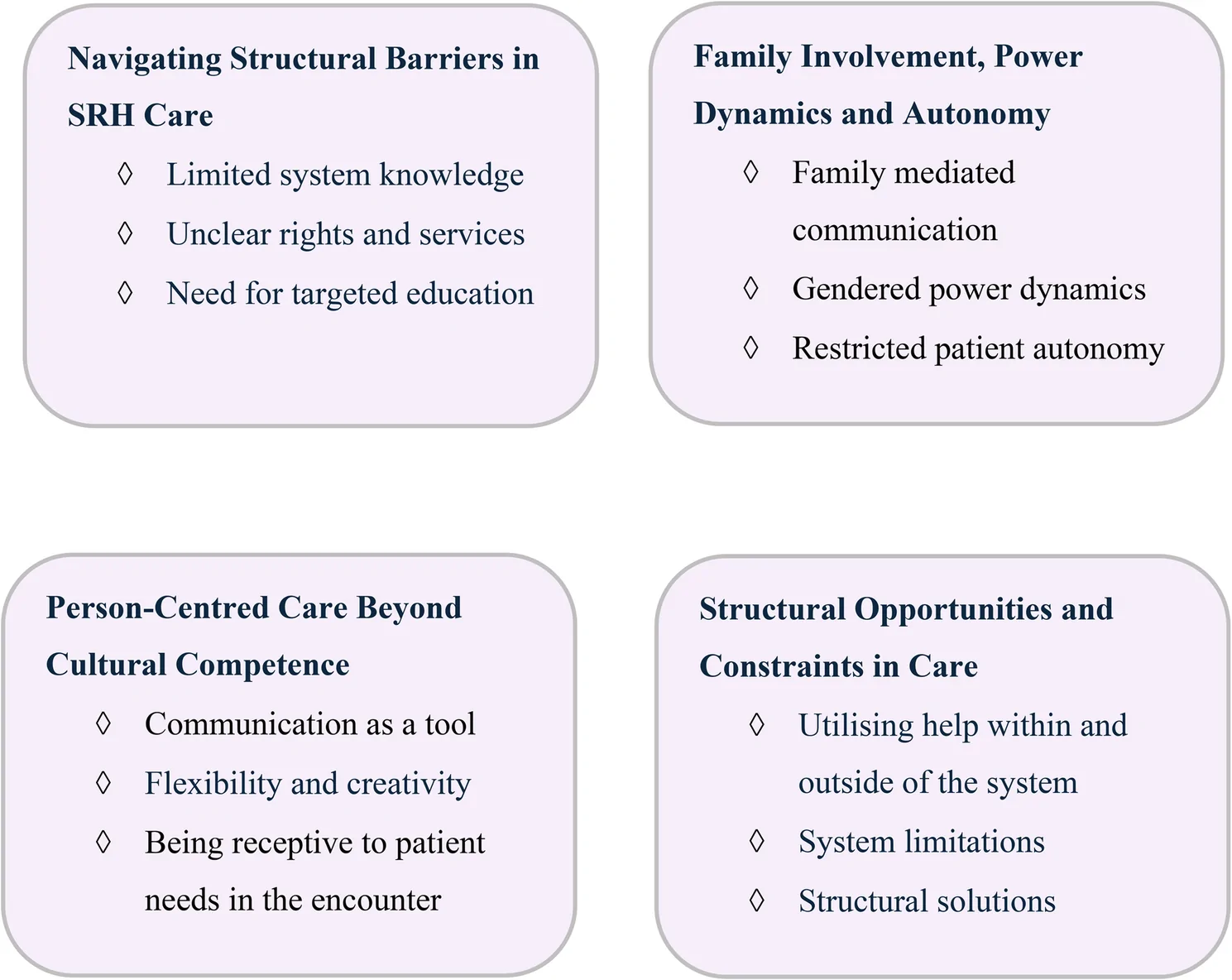
The resulting main themes from the results of factors influencing SRH within multicultural women´s health in Sweden

Throughout the interviews, participants referred to patients using a range of terms, including migrants, foreign-born individuals, and patients from multicultural backgrounds. In this study, these terms reflect the participants’ own language and perceptions. Unless otherwise specified, the findings relate to healthcare providers’ experiences of caring for patients whom they perceived as having migration-related backgrounds (Fig. 1).

### Navigating structural barriers in SRH care

A recurring theme in the interviews was navigating the health system and health literacy. Respondents described encounters with patients who come from countries where private healthcare is the norm and where it is normal to be able to choose the type of healthcare a patient wants, contrasting with Sweden’s universal model.

“I mean, there are women who come here from countries where healthcare is paid for and where you pay to have things done in a certain way, like paying for a C-section… Here, all healthcare is provided on equal terms, regardless of means.”

Participants described equality in access to care as a core principle of the Swedish healthcare system, while also noting that patients may arrive with different expectations regarding how healthcare is organised and delivered. Participants expressed the belief that many foreign-born patients nonetheless face knowledge barriers when trying to access SRH services. Participants described unfamiliarity with how the Swedish healthcare system is organised and how care is accessed and navigated, which, according to providers, could contribute to delayed care-seeking or inappropriate use of emergency services.

“I met a patient at the gynaecology clinic, who came from Congo. She came in with complaints of irregular bleeding, but when I delved deeper into her medical history, I realized that she was there because she had questions about both pregnancy, fertility, and sexuality. She did not really know which path to take, so she ended up at the gynaecology emergency unit, with bleeding issues.”

Even when patients reach SRH clinics, they may be unaware of available services. This highlights that although equality is central in the healthcare system, barriers arise creating inequities within the population. Respondents noted that difficulties in navigating the health system was especially common among older women, while younger women often access information through peers or social media.

“Older, non-Swedish-born individuals also don’t know what they can demand, compared to Swedish-born people… whereas a non-Swedish-born person of the same age does not really know what they are entitled to from healthcare. And I think that leads to very unequal care.”

This further highlights factors contributing to inequities in accessing and receiving care. To address these challenges, several participants emphasized the importance of educational initiatives aimed at improving patients’ understanding of the system.

“If there was some kind of education for them about the Swedish healthcare system… What rights they have, who they’re supposed to see, why they need to see a nurse before seeing a doctor….”

Several participants suggested that this type of education could be implemented in the SFI (Swedish for Immigrants) program. Internal staff training was also seen as vital for improving communication and cultural understanding.

“I strongly believe in the importance of internal training. One approach might be to engage with people who have received care, allowing them to share their experiences… How did they experience the treatment they received? Would they have preferred to be treated differently?”

These findings point to health literacy and system navigation as key factors in equitable SRH access. Targeted education, both for patients and providers, emerges as a concrete strategy to reduce disparities.

### Family involvement, power dynamics and autonomy

Family influence emerged as a central factor shaping women’s autonomy in SRH encounters. Providers described how family members, particularly male partners, but at times even children, acted as intermediaries during consultations, which limited opportunities for confidential communication. The use of children as interpreters was seen as especially problematic, as it prevented clinicians from addressing sensitive topics such as violence or coercion.

“Having a child serve as an interpreter in such situations can be highly problematic. It places the child in a position where they are not only exposed to intimate details but also expected to understand and convey them. We want to ask about threats or violence, but we cannot do that if there is another patient or a family member present.”

Providers described how they felt male partners could also dominate clinical conversations, answering questions on behalf of women, or discouraging them from speaking openly. These patterns were interpreted by participants as extensions of broader social and linguistic hierarchies within some families, where men in some contexts had more opportunities to engage with Swedish society and language.

“Men have, in many cases, been compelled in a different way to go out and use their Swedish, whereas women often have not had the same opportunities… But what I have noticed is that when their partner is not present, they actually become more confident, and they make use of their Swedish language skill more readily.”

Another dimension of family influence concerned the preference for female providers. While this preference is commonly expressed by female patients, both those with a Swedish and with a multicultural background, several participants perceived that male relatives sometimes made these decisions on behalf of the woman, occasionally requiring her to seek permission before accepting care.

“Yeah, it is very common, and it’s more common among people who weren’t born here. A lot of what we experience here is that it’s often the man who asks, “Is it a male doctor or a female doctor?” Or that if a woman is told she will see a male doctor she often has to call her husband and ask if it’s okay.”

Certain encounters were described where male partners invoked religious arguments to restrict women’s access to male clinicians. Providers also reported of rare but challenging encounters where husbands refused examinations entirely.

“A man refused to allow his wife to be examined… When we tried to reason with him, he insisted that the only acceptable condition would be for the healthcare provider to convert to his religion… then they could examine his wife”

Although there were accounts of family, especially male involvement, impacting individual autonomy, others highlighted positive aspects of this involvement.

“But then I think it might also just be about feeling safe. In many countries, people are more accustomed to having a family member present, whether that is a husband, mother, grandmother, or someone else who can provide support or help receive the information.”

Thus, relational involvement was also believed to facilitate and be important for the person seeking and receiving care. To navigate situations where being treated by a male doctor was perceived as an issue, seeking support from religious leaders was discussed, particularly Imams, who could clarify that male examinations are permissible in medical contexts.

“At the same time, there have been others of the same religion where it hasn’t been a problem at all. They have been informed that, and I believe that there has been some kind of agreement, because this has mostly involved Muslims, where the Region, the County Council, in consultation with an imam, has issued some kind of statement, that there are no obstacles, no religious barriers to being treated by a male doctor… when it comes to healthcare, the usual cultural, religious, or traditional norms don’t apply in the same way.”

Collaboration with religious leaders was described as a strategy used to facilitate care in situations where religious concerns were perceived to influence healthcare decisions. The example also highlights that although encounters were described in which the desire for a female doctor was motivated on religious grounds, in many cases, religion was not an issue. Providers also reported situations where linguistic concordance was considered more important than access to a female healthcare provider, as it was perceived to reduce the risk of misunderstanding. Other factors providers discussed as influencing the desire for a female provider was age and previous experiences. Finally, several respondents with international experience noted that such restrictions were often less pronounced in patients’ countries of origin.

“…I worked in Jordan for a year, and there, no one ever raised concerns about whether the doctor was a man or a woman. They were simply grateful that there was a doctor available. So, to me, it feels a bit surprising that patients who come from Jordan take issue with male gynaecologists here, when in Syria or Jordan, it’s completely normal to see a male doctor, and it doesn’t seem to be an issue”

The participant described experiences that suggested that expectations regarding gender and healthcare could be negotiated differently following migration and resettlement.

Power dynamics and family involvement were also discussed in respect to youths, especially female youths. Providers described encounters involving what they perceived to be honour violence and oppression, including concerns about early marriage and accounts of young women being sent abroad by their families. Family influence was also discussed in relation to contraception counselling.

“Some young women say they don’t want to use birth control pills because they are afraid their parents might discover it. Intrauterine devices are a possible solution. But there is also the contraceptive ring. It is not visible either, but the contraceptive patch is not an option, and the contraceptive injection is not recommended for younger women in general. Many have heard of the injection, but no, we try to explain why they should not use it.”

Respondents described how they believed concerns about negative parental or familial reactions could influence young women’s contraceptive choices. This was met by offering discretion in contraceptive counselling.

These accounts illustrate how providers perceive family dynamics and gender norms as influencing communication and the clinical encounter. This highlights how providers have to navigate multiple structural influences, such as social, gender norms, and religious, shaping the encounter while providing person-centred care. Providers described several strategies for navigating these situations, including speaking with patients privately, avoiding the use of family members as interpreters and, in some cases, involving community or religious leaders. Such involvement could help address religious concerns and facilitate access to care. However, it could also risk shifting influence over healthcare decisions away from the patient. The involvement of religious or community leaders should therefore be guided by the patient’s own wishes and should support, rather than replace, her informed and autonomous decision-making.

### Person-centred care beyond cultural competence

Participants described person-centred care as a central approach to addressing challenges that arose in SRH encounters. Rather than relying on assumptions about cultural background, providers emphasised the importance of understanding each patient’s individual circumstances, preferences, and social context. Participants described encounters in which patients were perceived to face considerable pressure from family or community members regarding personal health decisions. This was particularly evident in cases involving premarital sexual activity or abortion, where providers reported concerns that patients feared exclusion, stigma, or social repercussions.

“For example, I have been in a situation where I had to help a patient leave through the back exit so that she wouldn’t be seen by people as she left the women’s clinic. She had either undergone an abortion or had a miscarriage, and she knew she was being watched… And you also have to think about how to document it in the medical record and make sure that no letters are sent home to the patient that could be opened, or any text message.”

This example illustrates how providers adapted their practice to safeguard confidentiality and patient safety in response to an individual’s circumstances. This illustrates applying flexibility and creativity to minimise risks associated with disclosure. These accounts highlight how person-centred care extended beyond clinical consultations to include practical adaptations aimed at enabling patients to access care safely. Other examples involved adapting healthcare encounters to patients’ preferences and beliefs. Participants described modifying clinical approaches to respect religious values.

“Especially with some young Muslim women: when conducting an ultrasound, it is very important for them that nothing is ruptured. In these cases, it is often suggested to perform a transrectal ultrasound, which works very well. You get a very good image with a transrectal ultrasound, and most women are willing to accept it.”

This account reflects the participant’s description of some patients’ concerns about preserving hymenal integrity. However, hymenal appearance is not a medically valid indicator of sexual history. The example therefore illustrates an ethical tension in person-centred care: adapting the examination may respect an individual patient’s expressed preference and protect her from potential social consequences, while such accommodation may also risk reinforcing restrictive beliefs surrounding female sexuality and perceived virginity. Providers must therefore navigate between responding to the individual patient’s circumstances and avoiding the legitimization of beliefs that may constrain sexual and reproductive autonomy.

Providers also reported encounters in which they reframed contraceptive prescriptions as medical treatments with the aim of reducing stigma and gaining parental acceptance.

“We try to explain and de-dramatize these treatments, especially when prescribed for issues like severe menstrual pain rather than sexual activity. If a doctor, wearing a white coat, explains the medical necessity of the prescription to the parents, it often gains legitimacy… Sometimes, I can offer to explain to the parents that the prescription is for medical reasons, not related to sex.”

In this example, participants described adapting communication and framing contraceptive use in ways they believed would facilitate acceptance and access to care. These accounts illustrate how providers sought to navigate situations in which they perceived familial or social concerns to influence healthcare decisions. Participants described using flexibility and professional authority to support patients’ access to SRH services while remaining attentive to the individual’s circumstances.

While broader research focuses on group-level challenges, these accounts show how providers offer individualized, empathetic care in complex social contexts.

“I have to try to… adjust myself to the level that I think the woman can take in, in what I say, what I explain, or what I… want to convey. Just like with Swedish born women, you have to adapt to the person in the room. Where she is at. And this isn’t only about where she is in life, but also in her everyday situation. Yes, education level and knowledge level can vary among Swedish born women too. So you always have to be attentive to that in some way.”

Participants describe using communication as a central element of person-centred care, taking into consideration the patients individual background and situation in all clinical encounters. Several participants highlighted that such variation existed across all patient groups and was not unique to patients with migration-related backgrounds.

The importance of supporting patients without imposing personal values was underlined while maintaining a respectful and professional stance.

“Well, I think I do that by… always respecting what my patients think and feel. And responding to that as well as I can. You’re only human, of course, but… being open to the fact that people think differently, grew up differently, that we all have different backgrounds, being open to that… Even though you’re supposed to provide equal care to everyone, in some way, yes.”

This account describes the tension between equality and equity in care, with the provision of equal care for all patients being central to healthcare provision in Sweden, while also highlighting factors that require care to be individually adapted to patients’ circumstances and needs.

Ultimately, participants emphasized the need to balance respect for cultural belonging with efforts to empower patients.

“My ambition is not to persuade anyone, but rather to show the possible paths available… I am not supposed to impose my values on her, but rather to try to find out where she is….”

This reflects a strong example of complex professional navigation using communication as a tool to provide person-centred care. Providers draw on clinical experience and flexibility to adapt their approach to each unique patient and situation, acknowledging the intersecting cultural, religious, educational and social influences on the encounter. As some respondents suggested, such person-centred adaptability is essential in clinical practice, where a one-size-fits-all approach is rarely effective.

### Structural opportunities and constraints

Participants described many structural challenges and solutions in providing sexual and reproductive healthcare to migrants. The issue of upholding confidentiality was broached, especially regarding parental access to digital records. It was perceived that migrant youth were more likely than Swedish-born peers to inquire about protecting their information.

“It is possible to log in using Bank-ID to access one’s medical records after a child has turned 13 years old. And parents can do this if they have access to their child’s Bank-ID. We often receive questions about who can read their medical records… and how one can block or protect their medical records. These questions mainly come from migrant youth. Swedish- born adolescents rarely ask about this… There are also those who say that every Friday after school, they must hand over their phone to their parents for the weekend. The parents then have access to everything, messages, browsing history, and Bank-ID. In such cases, they can even log in and read the child’s medical records.”

This example illustrates how confidentiality risks emerged through the interaction between family circumstances and digital healthcare systems. Rather than being solely interpersonal, participants described these challenges as shaped by institutional and technological structures.

Additionally, even though medical records and prescriptions are supposedly confidential and, for those who need them, paper prescriptions are issued, confidentiality can still be compromised through digital pharmacy platforms, deterring youth from seeking SRH care.

“Even though we have systems in place to protect confidentiality, like hidden medical records or blocking access via 1177, issues still arise. For example, prescriptions from some online pharmacies can clearly display that a minor has been prescribed contraceptives, such as birth control pills. There is currently no regulation preventing parents from logging in and discovering this information. This kind of unintended openness can be harmful.”

Despite the vision of increasing patient autonomy and inclusion in care by having access to medical records digitally, these examples highlight untended side-effects which could comprise access to care. Another example participants had of digitalisation creating structural barriers to care is the use of online consultations.

“[The] online app, UM Online… Not all young people’s BankIDs even work with it. I think we’ve closed the door to a great many people there, particularly unaccompanied young people who may not have anyone to help them with it.”

This was reported to be particularly consequential during the COVID pandemic where the majority of consultations at youth clinics were online. As this example illustrates, already vulnerable groups, such as unaccompanied young people, were believed to be more greatly impacted. Many of these structural barriers can be difficult to mitigate yet providers shared strategies for discreet communication and recordkeeping to address risks relating to confidentiality.

“I always inform them of their rights, and there are also phone numbers that appear neutral when dialled… And in especially sensitive cases, such as when a patient is hiding from a violent partner or a family structure that is abusive, we maintain a physical, handwritten record of their contact details… This ensures we have a discreet way of reaching them.”

This example highlights both individual and institutional solutions to situations which require particular discretion on part of the health system to ensure patient confidentiality and safety. An example of a structural adaption was the relocation of clinics to areas with more diverse populations to improve access to care.

“We relocated our clinic, and we deliberately chose the southern part of the city, which is significantly more multicultural than the north… many young people with immigrant backgrounds go to school or move about in that area. And to be frank, we thought that those born in Sweden would manage to find their way there without issue. Following the move, we did see an increase in patients from foreign backgrounds.”

This example illustrates how participants described efforts to improve access through adaptations at the organizational level. Rather than focusing solely on individual encounters, providers also highlighted how the location and organization of services could influence whether young people felt able to seek care.

In clinical encounters, participants similarly described drawing on available resources within the healthcare system to support patients and promote informed decision-making.

“I often refer them to a counsellor, and I remind them that this is Sweden, where we have laws that safeguard gender equality, that she alone has the right to make decisions about her body, her fertility, and her life. There is absolutely no law that permits anyone to take that right away from her.”

Providers described making individual assessments based on each patient’s circumstances and, when needed, connecting them with additional support services.

“And what I was also able to do… was to connect her with the social services team that works with honour-related issues. She was living in an honour culture…. Had this come to light, she would most likely have been thrown out of her home since she lived with her family… she said, ‘No, not physical violence, but I would no longer be part of the family; they would exclude me from the community.”

In this example, the provider described drawing on support structures outside the healthcare system in response to concerns about social exclusion and control within the patient’s family environment. Across the interviews, participants highlighted the importance of using resources both within and beyond healthcare to support patients facing complex social circumstances. These accounts illustrate how providers not only navigated structural barriers but also sought to mobilise institutional and community resources to promote equitable access to care.

## Discussion

This study illuminates how sexual and reproductive health providers navigate multiple intersecting influences while striving to deliver equitable care to patients from multicultural backgrounds. Participants described balancing individual preferences, family relationships, social norms, professional obligations, and structural constraints, while demonstrating a strong commitment to autonomy, confidentiality, and equitable access to care.

### Providing equitable SRH care requires navigating multiple intersecting influences

Our findings suggest that providing equitable SRH care in multicultural settings involves navigating multiple intersecting influences rather than addressing culture alone. Providers described balancing individual patient preferences, family relationships, social norms, religious beliefs, safety concerns, professional responsibilities, and legal obligations, often within the same clinical encounter. Culture was rarely treated as a fixed explanatory factor; instead, participants emphasized understanding each patient’s unique circumstances and adapting care accordingly.

Providers reflected on how patients bring diverse experiences and backgrounds into the healthcare encounter and described tailoring their approach to the individual rather than relying on assumptions based on religion, ethnicity, or presumed cultural affiliation. This aligns with previous research demonstrating that assumptions about patients’ preferences can contribute to stereotyping and poorer care experiences [4, 26, 36], Participants recognised culture as one of several factors shaping healthcare encounters and described an ongoing process of reflection and adaptation. These findings resonate with concepts such as cultural humility, which emphasize self-reflection, attentiveness to power dynamics, and openness to patients’ individual experiences, as well as structural competency, which highlights how broader social and institutional conditions influence health and healthcare encounters [26, 27].

The complexity of this work became particularly visible when providers described balancing patient autonomy with family expectations, religious beliefs, and social norms. Participants highlighted situations in which patient preferences did not always align with assumptions about cultural or religious backgrounds. For example, While some patients prefer female providers, other studies show that competence and safety are often the primary determinants of provider choice, and some patients even prefer male providers who share language or cultural background to avoid perceived discrimination [37]. These examples illustrate the complexity of SRH encounters and the competing considerations that clinicians must navigate when balancing patient preferences, autonomy, safety, and equitable access to care.

Participants described involving religious leaders to clarify that religious teachings do not necessarily prohibit care by male clinicians. While such involvement may facilitate communication and access to care, it may also raise ethical concerns if external perspectives influence individual healthcare decisions. Our findings therefore highlight a tension between culturally responsive care and safeguarding patient autonomy [12, 19, 38, 39]. Any involvement of religious or community figures should be guided by the patient’s informed preferences and established medical, ethical, and legal principles [4]. This raises questions about legal and institutional boundaries and the need for clearer frameworks to reconcile person-centred care with statutory obligations, that also have been brought up in earlier studies [23, 24].

Crucially, providers described navigating not only cultural expectations but also the broader social, structural, and normative systems in which these expectations are embedded. Many young women moved within and between these intersecting systems, and providers emphasized the importance of meeting them in their specific circumstances without moral judgement. This flexible and individualized practice suggests that equitable multicultural SRH care is less about managing cultural difference and more about navigating the complex interplay of individual, familial, social, and institutional influences that shape patients’ opportunities to exercise autonomy and access care. We believe this to be a key discovery that should inform policy and guideline development. Listening to providers’ insights will help ensure that legislation supports safe and equitable SRH care for all patients, regardless of cultural background [10, 12, 27].

### Equitable care is shaped by structural conditions, not only clinical practice

While providers described considerable flexibility and creativity in responding to patients’ needs, the findings indicate that equitable care is also shaped by the health system structures within which care is delivered. Participants identified organizational, legal, and digital factors that influenced access, confidentiality, and autonomy, highlighting that equitable care cannot rely solely on individual clinicians’ efforts.

A key tension emerged between providers’ ethical obligations to individual patients and the structural constraints of the Swedish healthcare system. Swedish healthcare is governed by laws such as the Swedish Health and Medical Services Act (Hälso- och sjukvårdslagen) [40] and the Patient Act (Patientlagen) [41] both of which emphasize equal access and equitable treatment. However, participants frequently described situations in which achieving these aims required extending beyond formal routines, a practice that points to the importance of institutional support, clearer guidance, and better patient education about the healthcare system [5, 38, 42]. Patients’ opportunities to access care, maintain confidentiality, and exercise autonomy were influenced not only by clinical interactions but also by organizational arrangements, legal frameworks, and digital infrastructure. These findings suggest that education about sexual and reproductive health and the Swedish healthcare system is important but may be insufficient if structural barriers remain unaddressed [10–12].

Participants also highlighted several examples of how healthcare structures can both facilitate and hinder equitable care. Structural adaptations included relocating youth clinics to areas with more diverse populations, utilising referral pathways to counsellors and social services, and creating mechanisms to protect confidential health information for particularly marginalised patients. At the same time, participants identified systemic vulnerabilities that could undermine confidentiality and access. Digital systems, including pharmacy platforms and electronic access to medical records, were described as creating confidentiality challenges for some young people living in controlling family environments, where privacy could not always be assumed. These findings highlight how digitalisation, although often intended to improve access and efficiency, may also create unintended vulnerabilities for patients whose autonomy is constrained by family or social control. Confidentiality therefore extends beyond the clinical encounter itself and is also shaped by the design of health-system infrastructure and digital services.

Many of the strategies described by providers can be understood as responses to these structural gaps. Participants reported drawing on their clinical experience to navigate barriers when family control, honour-related violence and oppression, or concerns about confidentiality limited access to care. While such flexibility in ethically complex encounters, rather than rigid adherence to guidelines, reflects professional commitment and adaptive practice, a pattern that aligns with earlier work on adaptive professionalism in SRH care [24, 27], the findings suggest that equitable care should not depend on individual clinicians identifying solutions on a case-by-case basis. Instead, the need for such workarounds highlights how organizational, legal, and digital systems can both enable and constrain equitable care. Rather, healthcare systems must actively support confidential communication, patient autonomy, appropriate referral mechanisms, accessible health information, and privacy-sensitive digital infrastructure. These should include provider training and technical safeguards, so that protection for marginalized groups does not depend solely on individual clinicians’ flexibility and generosity, in line with previous studies [11, 27, 38]. The findings therefore underscore the importance of incorporating frontline providers’ perspectives into policy development and service design. Participants’ experiences reveal how legal, organizational, and technological arrangements can either enable or constrain equitable care in practice. Strengthening these structures may reduce reliance on individual workarounds and help ensure that safe, confidential, and person-centred SRH care is consistently available to diverse patient populations.

Rather than highlighting cultural challenges in isolation, this study demonstrates that providers navigate the intersection of individual preferences, family relationships, social norms, professional obligations, and institutional structures. Equitable multicultural SRH care therefore requires both person-centred clinical practice and health systems that actively support confidentiality, autonomy, and access.

### Strengths and limitations

This study draws on interviews with a diverse group of SRH professionals, including physicians, midwives, nurses, and social workers, which enabled a multifaceted analysis of care practices in multicultural settings. Variation in professional background, gender, and migration history strengthened the analytical depth. Conducted in Skåne, particularly Malmö, one of Sweden’s most demographically diverse regions, the study is well situated to explore SRH care in relation to migration and integration. Participants’ extensive clinical experience with multicultural populations further supports the credibility of the findings.

Analytical rigor was enhanced through interdisciplinary triangulation.

Several limitations should be considered. The findings reflect providers’ perspectives and cannot establish migrant patients’ experiences or motivations. The interview guide focused on challenges and dilemmas, which may have emphasized problematic encounters and contributed to a deficit-oriented representation of migrant patients. Where supported by the data, we therefore also highlight patient agency, supportive family involvement, successful encounters, and instances where providers’ assumptions were challenged. Convenience and snowball sampling, including recruitment through professional networks, may also have introduced selection bias.

The dataset has been used in previous publications addressing different research questions and involving partly different analytical teams; some conceptual overlap is therefore inevitable. Data were collected during the COVID-19 pandemic, and some organizational and digital practices may have since changed.

## Conclusion

This study highlights how SRH professionals perceive and navigate the interplay of individual, familial, social, and structural factors in multicultural clinical encounters. Providers described using flexible, person-centred approaches to support autonomy, confidentiality, and access to care while navigating family dynamics, differing social and religious norms, and institutional constraints. Their accounts suggest that equitable SRH care should not depend primarily on individual clinicians adapting or improvising solutions to structural barriers. Healthcare systems should support confidential communication, appropriate interpretation, patient-centred decision-making, protection from coercion, accessible health-system information, appropriate referral pathways, and privacy-sensitive digital infrastructure. Incorporating frontline providers’ experiences into service development, alongside continued professional training and clearer institutional guidance, may reduce reliance on individual workarounds and strengthen equitable, person-centred SRH care. Future research should incorporate migrant patients’ perspectives and examine how structural adaptations influence access, confidentiality, autonomy, and equity in care.

## Data Availability

The datasets used and/or analysed during the current study are available from the corresponding author on reasonable request.
